# Book Review

**Published:** 1932

**Authors:** 


### Reviews of Books

**Choosing a Wife and Other Essays.** By E. G. Dru Drury, M.D. Pp. viii, 279. London: H. K. Lewis & Co. Ltd. 1932. Price 8s. 6d.—This is a collection of three groups of papers and addresses delivered in South Africa to medical students, nurses and fellow-practitioners respectively. The subjects are varied and none of the discourses are very technical. For the most part they make pleasant reading—highly entertaining in the case of many, especially the student group. Although the addresses to the nurses are more or less educational, the author introduces into most of the other essays a surprising number of facts, anatomical, genetic, physiological and clinical, which appear as similes or examples in the philosophy he unfolds. Two of the addresses, " Grahamstown Hospitals " and " Medical Trades Unionism," afford glimpses of local history. The latter, dated 1906, is of some interest in view of the subsequent events in South African medical politics. The style throughout is interesting. In places one seems to be reading whole paragraphs of pithy aphorisms which, however, do not pall, and homely examples, among which the motor-car frequently figures, are often used in a telling way to drive a point home. One might wish for such a teacher.

**A Guide to General Practice.** By A. H. Douthwaite, M.D. Pp. viii, 96. London: H. K. Lewis & Co. Ltd. 1932. Price 4s. 6d.—The author is to be congratulated on having produced in so small a space and in so readable a manner so much information, advice and warning. In five years' experience of general practice he has obtained an insight into life, as a doctor encounters it, that might have been expected in forty years, and in general it is a view with which probably most medical men of much experience will agree. The reader should begin with the first two paragraphs of " Conclusion," otherwise he may get an impression that the author has struck an unusually bad patch. It is a book that should be studied carefully by every man starting practice, as a valuable education that otherwise he could only get by hard experience; it should save him much unhappiness; and it may be read with enjoyment by older men as a humorous, if sometimes cynical, reminiscence. There is a paragraph on vampire-like parents that deals, perhaps a little hardly, with a subject that must have caused most men anxiety, and opens up a question of considerable social importance that wants more consideration than it has received. We read with interest and agreement his remarks on the gullibility of the educated classes.

**Methods and Objective of Medical Education.** Part II of a Report to the Government of Victoria on the Hospital Medical School Problems of the State of Victoria. By R. J. A. BERRY, M.D., F.R.C.S., F.R.S. Published by the Government of Victoria. 1929.—Professor Berry was appointed by the Government of Victoria to visit the hospitals and medical schools of the United States, Canada, and England, and this is one of several most interesting and valuable reports that he made. It might well be called "Through the Schools with a Seeing Eye." At the present moment, when the question of Medical Education is so much to the fore, this memorandum is particularly valuable. As a professor of anatomy and dean of a medical school, the writer speaks with authority. With his well known insight and logical method, and a pleasant touch of humour, he describes, discusses and contrasts the educational systems of the United States, Canada and Britain. He has much of practical interest to say on preliminary study, selection of students, training for specialist versus training for general practitioner. He discusses the value of lectures, the means and methods of research, which seem to be unlimited in the States. He has an open mind on most questions, and says that we have much to learn from America, especially in its method of administration, which is much superior to ours. The Report is one that should be read by everyone interested in the question, and it is to be hoped that copies may be available, though it was published in Melbourne three years ago.

**Baillière's Synthetic Anatomy.** By J. E. CHEESMAN. 156 plates. London: Baillière, Tindall & Cox. 1932. Price 42s.—This work consists of a series of superimposed anatomical plates, so arranged that the part in use can be built up, and also dissected, by placing or displacing transparent constituent sheets one on the top of the others. While this method of study may not appeal to many, and will certainly in no way replace the study of Anatomy by more orthodox methods, in lecture-room and dissecting-room, it may well be that it will be found useful for revision and for self-examination. For those to whom this method appeals, nothing more suitable than these plates can be imagined. They are clearly drawn and anatomically accurate, and although the method of study is apt to be confusing on first acquaintance, it tends to grow on one as its advantages are appreciated in use. The series under review can be cordially commended to those for whom it has been designed.

**Cancer: Civilization: Degeneration.** By J. COPE. Pp. xvii, 293. Illustrated. London: H. K. Lewis & Co. Ltd. 1932. Price 15s.—This is another of those tedious sermons on the text "Back to Nature." The author claims to be a medical man employing a *nom de plume* for *ethical* reasons. It is difficult to believe that he has any clinical acquaintance with cancer: his object is "to make your flesh creep," and for this purpose he supports his *ex parte* statements by quotations from any and every source, without reference or context. The book abounds in such gambits as "many leading authorities consider," "there is an ever increasing conviction that," "all the evidence tends to show," whatever "fact" may be necessary for the argument of the moment. Appeal is manifestly to the ignorant and credulous, fortified with statistics from the Registrar's returns and seasoned with the philosophy of Freud. To illustrate racial degeneration we are shown the figures for physical fitness of *civilians of military age in* 1918! The predisposing causes of cancer are race degeneration, constipation, modern food (preserved, "prepared," lacking in roughage), indolence and luxury, avoidance or limitation of child-birth and nursing: even more than the effeminate man the author dislikes the sporting woman, unsexed, perverted, physically unattractive. He has not noticed that the "boyish" figure ceased to be worn even earlier than the ultra-short skirt, and forgets that his Golden Epoch at Athens produced, among other glories, "The Suffragettes." The exciting cause of cancer may be a blow, exposure to rays (including sunlight—but why not "wireless"?), finger rings, inhalation of petrol fumes, the toxin of influenza, alcohol "the toxin of the yeast bacillus," etc. The book is illustrated by a considerable number of rather crude pen and ink sketches: the author should learn anatomy before offering instruction in that subject. Despite a few misprints ("cararrh") the typography is worthy of a better theme.

**Plastic Surgery of the Nose, Ear and Face.** By VICTOR FRÜHWALD. Pp. viii, 86. Illustrated. Vienna: Wilhelm Maudrich. 1932. Price $4.—The object of this book is to refresh the memory of those who have attended the author's clinic in Vienna. He points out that "to acquire a knowledge of plastic surgery from this book is not possible," and one can only commend the good sense of this pronouncement. The subjects discussed are nasal plastic repair, minor aural deformities, removal of wrinkles, "face-lifting," etc. The very limited class for whom the book is designed will perhaps tolerate the irritating foreign constructions, etc., and, of course, will not expect the work of any English or French surgeons to be acknowledged. The illustrations are numerous and include many pictures of quite ordinary knives, chisels, etc. Some of them are incomprehensible (except, we suppose, to the chosen few).

**A Practice of Medicine.** By A. STRÜMPELL AND E. SEYFARTH. Translated from the Thirtieth German Edition by Marshall & Ottley. London: Baillière, Tindall & Cox. 1931. 3 volumes. Price £5 5s.—With the exception of infectious diseases and diseases of metabolism the classification employed in this book is mainly anatomical, and in the index, main and sub-headings are used with advantage. A good alphabetical index is also included in the third volume. The first section of the first volume is devoted to infectious diseases. Special mention in this section must be made of the excellent coloured plates and of the pulse and temperature charts typical of each disease, which should prove of practical assistance. It is, however, a little surprising to find leprosy included as an "acute infectious disease." The remainder of this volume deals comprehensively with diseases of the respiratory and circulatory systems and includes many well-reproduced radiograms. Exception might be taken to the statement on page 704 that "puncture of the pericardium is less dangerous than might be feared. Experience has shown that even if the heart itself is wounded serious consequences scarcely ever occur." It is also unfortunate that the drugs advised and discussed at length are often in unfamiliar form Diseases of the digestive and urinary systems are considered in the first three-quarters of the second volume. These sections are so well written that one hesitates to call attention to certain omissions. On page 918 "constipation" is given as one of the essential signs of acute appendicitis, whereat she increase in pulse rate is not mentioned. "Acute necrosis of the pancreas" is differentiated from "acute pancreatitis," but no reference is made to cyanosis in either. A short consideration of abdominal actinomycosis might have been added for the sake of completeness. The remainder of this volume deals with diseases of the organs of motion and of metabolism. The section on blood diseases also deserves special mention, although the existence of acute lymphatic leukæmia as a pathological entity may still be doubted by a few authorities. The last volume deals at length with diseases of the central nervous system, and this is the best section in the book. We have nothing but praise for the arrangement, and such lists as the segmental supply of muscles and the action and nerve supply of muscles should prove very useful to the busy practitioner. There are two appendices: the first a "summary of the more important poisons," and the second "a few useful prescriptions." A short review of so large a text-book must necessarily be incomplete, but after a study of this book, we can confidently recommend it to general practitioners. It is clearly printed on good paper and reads easily. Dr. Marshall and Miss Ottley are to be congratulated on their translation.

